# Value of the Safety Management System (VMS) frailty instrument as a frailty screener in care for older hospital patients: a systematic review

**DOI:** 10.1007/s41999-024-00957-4

**Published:** 2024-04-26

**Authors:** Frederike M. M. Oud, Meggie D. Meulman, Hanneke Merten, Cordula Wagner, Barbara C. van Munster

**Affiliations:** 1 Universitair Medisch Centrum Groningen, Groningen, The Netherlands; 2 Department of Geriatrics, Gelre Ziekenhuizen, Apeldoorn & Zutphen, The Netherlands; 3 Netherlands Institute for Health Services Research (Nivel), Utrecht, The Netherlands; 4 Department of Public and Occupational Health, Amsterdam UMC, Vrije Universiteit Amsterdam, Amsterdam Public Health Research Institute, Amsterdam, The Netherlands

**Keywords:** VMS frailty instrument, Vulnerable, Frailty, Older patient

## Abstract

**Background:**

Dutch hospitals are required to screen older patients for the risk of developing functional decline using the Safety Management System (VMS) which assesses four domains associated with functional decline; fall risk, risk of delirium, malnutrition, and physical impairment.

**Purpose:**

The aim is twofold, first to compare the VMS frailty instrument as a frailty screener with existing frailty instruments and second to provide an overview of the available evidence.

**Methods:**

We performed a literature search to identify studies that used the VMS instrument as frailty screener to asses frailty or to predict adverse health outcomes in older hospitalized patients. Pubmed, Cinahl, and Embase were searched from January 1^st^ 2008 to December 11th 2023.

**Results:**

Our search yielded 603 articles, of which 17 studies with heterogenous populations and settings were included. Using the VMS, frailty was scored in six different ways. The agreement between VMS and other frailty instruments ranged from 57 to 87%. The highest sensitivity and specificity of VMS for frailty were 90% and 67%, respectively. The association of the VMS with outcomes was studied in 14 studies, VMS was predictive for complications, delirium, falls, length of stay, and adverse events. Conflicting results were found for hospital (re)admission, complications, change in living situation, functional decline, and mortality.

**Conclusion:**

The VMS frailty instrument were studied as a frailty screening instrument in various populations and settings. The value of the VMS instrument as a frailty screener looks promising. Our results suggest that the scoring method of the VMS could be adapted to specific requirements of settings or populations.

**Supplementary Information:**

The online version contains supplementary material available at 10.1007/s41999-024-00957-4.

## Key summary points

**Purpose:**

The aim of this systematic review is twofold, first to compare the VMS frailty instrument as a frailty screener in older patients in different hospital settings with existing frailty instruments and second to provide an overview of the available evidence.

**Key findings:**

The VMS frailty instrument is used as a frailty screening instrument in various populations and settings. The VMS frailty instrument is predictive for adverse outcomes and has similar (reasonable) measurement properties as existing frailty tools.

**Message:**

The value of the VMS instrument as a frailty screener looks promising, the scoring method of the VMS could be adapted to specific requirements of settings or populations and aim of the screening.

**Supplementary Information:**

The online version contains supplementary material available at 10.1007/s41999-024-00957-4.

## Introduction

Hospitalized older patients are at risk of adverse events, such as functional decline, readmission or mortality, especially those who are frail [1–5]. Frailty is a medical condition of increased vulnerability and poor resolution of homeostasis after a stressor event as a consequence of cumulative decline in many physiological systems [6]. Identifying frail patients is important to prevent poor health outcomes, guide clinical decision-making, and advance care planning. For optimal management of frail patients, it has been suggested that all individuals over the age of 70 admitted to the hospital should be screened for frailty [7, 8]. Consensus on a frailty screening tool has, however, yet to be reached [7–9].

The Safety Management System (VMS) frailty instrument is used to screen older patients in Dutch hospitals to prevent or reduce functional decline [10–14]. The VMS was originally developed and gradually implemented in all Dutch hospitals from the year 2009 [11]. The VMS frailty instrument is a short mandatory questionnaire in the Netherlands aiming to identify older patients (aged 70 or older) at risk for delirium, falls, malnutrition, and physical impairment, consisting of 13 questions that do not require additional resources (Supplementary A, Fig. A.1). Delirium risk is assessed with three questions about: memory problems, previous delirium or confusion, and help with Activities of Daily Living (ADL). Fall risk is assessed with a single question about whether a patient has fallen in the past 6 months. Malnutrition is scored with either the Short Nutritional Assessment Questionnaire (SNAQ) or Malnutrition Universal Screening Tool (MUST). [15, 16] Physical impairment is scored with the six-item Katz Index on Independence in ADL [17].

**Fig. 1:**
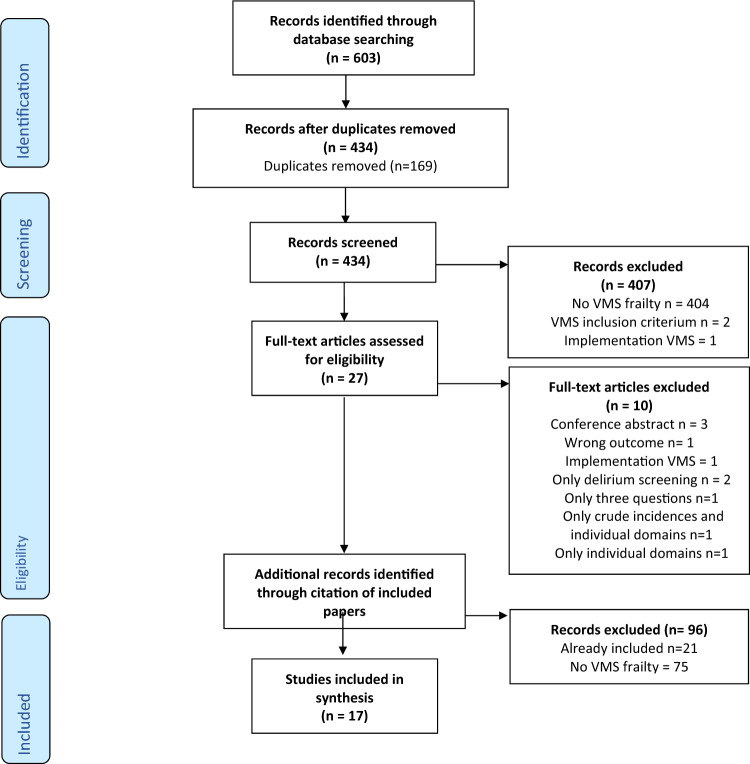
Flowchart of included and excluded studies

Because the VMS frailty instrument was not originally designed as an integrated frailty instrument, a cut-off for frailty was not established before introduction. Lately, there is a growing number of studies using the VMS frailty instrument to identify frailty, rather than assessing the individual domains to prevent or reduce functional decline in older patients. These studies focus on different hospital settings, populations, and outcomes with a variability in cut-off scores in clinical practice and research. Since Dutch hospitals are required to screen all patients aged ≥ 70 years with the VMS frailty instrument, it can be of added value if the VMS frailty instrument can also predict frailty or adverse events in older patients. Besides, the VMS frailty instrument may also be of interest outside the Netherlands, as included instruments (e.g., fall risk question, SNAQ, MUST, Katz-ADL6) are internationally validated instruments.

To this day, the predictive value of the VMS frailty instrument as a frailty screener and the diagnostic test accuracy of various cut-off scores are still unknown. Therefore, the aim of this systematic review is twofold, first to compare the VMS frailty instrument as a frailty screener in older patients in different hospital settings with existing instruments and second to provide an overview of the available evidence to be able to make a recommendation for future use.

## Methods

This review was conducted and reported following the Preferred Reporting Items for Systematic reviews and Meta-Analyses (PRISMA) Checklist. (Supplementary B). The software used for the management of the results of the search was Rayyan [18].

### Search strategy and selection criteria

Electronic bibliographic database searches were performed in Pubmed, Embase, and Cinahl. The search strategy was designed in consultation with an information specialist. We searched with the terms aged, older, and VMS, safety management and screening. A detailed description of the search strategy is provided in Supplementary C. We checked the citations of included articles for other eligible studies that were not found in the search. We executed our search in December 11th 2023.

Two researchers (FO and MM) independently performed article selection based on title and abstract against predefined inclusion criteria, and if an article was deemed potentially relevant, they assessed the full text on study eligibility. Inclusion criteria were: 1) the VMS frailty instrument with a cumulative or cut-off score must be examined as an instrument to predict frailty or adverse health outcomes in older patients, 2) articles had to be published in English or Dutch. We did limit on publication date (from 2009, because from here the VMS was implemented), but not by study design, setting, or outcome measure. Studies that only used the VMS frailty instrument to identify or describe frail population were excluded. Studies that did not cover all four individual domains in the VMS frailty instrument were excluded.

Details of included studies are given (Table 1). For excluded sources; reasons were stated on why they were excluded (Fig. 1: flow chart).

**Table 1:** Characteristics of included studies

	References	Design and number of participants	Population	Gender and age	VMS frailty score	Outcomes	Length of follow-up
1	Calf 2020 [22]	Prospective cohort 196	ED* patients ≥ 65 years	Median 72.5 (IQR 68–78) Male 51.5%	Aged 70–80 ≥ 3 Aged ≥ 80 ≥ 1	Frailty (frailty question) Mortality	1 year
2	Cords 2023 [31]	Retrospective cohort Multicentre 129	Burn injury patients ≥ 70 years	Mean 65.8 (SD 11.5) Male 40.2	Number of domains	Frailty (CFS)□	n/a
3	Heim 2015 [13]	Prospective cohort 883	Hospital patients ≥ 70 years	Median 79 (IQR 9) Male 46%	Aged 70–80 ≥ 3 Aged ≥ 80 ≥ 1	Frailty *(ISAR-HP) ∪ * Adverse outcome (functional decline, high-healthcare demand, death)	3 months
4	Hermans 2019 [26]	Prospective cohort 206	Patients who underwent PCI† for STEMI‡ ≥ 70 years	Mean 79 (SD 6.4) Male 85%	VMS score ≥ 1 (0–4)	Mortality PCI-related SAEs§ (clinical death, target vessel failure, major bleeding, contrast induced kidney insufficiency and stroke)	30 day
5	Oud 2015 [14]	Prospective cohort 688	Hospital patients ≥ 70 years	Median 78 (70–79) Male 50.7%	Number of domains	Mortality	6 months
6	Oud 2021 [39]	Retrospective cohort 477	Geriatric inpatients	Median 85 (54–99) Male 37%	Number of domains VMS score ≥ 1 (0–4)	Mortality Institutionalization Readmission	3 months 12 months
7	Oud 2022 [30]	Retrospective cohort 4478	Hospital patients ≥ 70 years	Median 79 (70–101) Male 44.8%	Number of domains	Mortality	3 months
8	Schuijt 2020 [20]	Prospective cohort 249	ED patients ≥ 70 years	Median 80 (IQR 75–86) Male 39%	Number of domains VMS score ≥ 2 (0–4)	Mortality Falls Readmission ED readmission Functional decline Change living situation Composite outcome (either death or functional decline (loss of points on KATZ-ADL)	90 dayss
9	Snijders 2020 [24]	Retrospective cohort 448	ED patients ≥ 70 years	Median 77 (IQR 73–82) Male 50.7%	Short-VMS|| ≥ *2 (0–5)*	Mortality ED readmission Hospital admission	90 day
10	Souwer 2019 [25]	Prospective cohort Multicentre 550	Colorectal cancer surgery ≥ 70	Median 76.5 (﻿IQR 74–82) Male 53%	Low risk = 0; intermediate risk = 1 and 2, high risk = 3 and 4	Complication (surgical, non-surgical, cardiopulmonary, any) Delirium Readmission Survival	Median 870 days
11	Van Dam 2018 [29]	Prospective cohort 265	ED patients ≥ 70 years	Median 76 (IQR 71–81) Male 50%	Aged 70–80 ≥ 3 Aged ≥ 80 ≥ 1	Frailty *(ISAR-HP) ∪ * Hospital admission	n/a
12	Van Dam 2021 [40]	Prospective cohort 889	ED patients ≥ 70 years	Median 78 (IQR 73–78) Male 48%	Aged 70–80 ≥ 3 Aged ≥ 80 ≥ 1	Functional decline Institutionalization Mortality	3 months 6 months
13	Van Loon 2017 [28]	Prospective cohort 123	(Pre)dialysis ≥ 65 years	Mean 76 Male 64%	VMS score ≥ 2 (0–4)	Frailty (CGA#, ISAR-HP ∪ , frailty question, GFI ~ , FFI ∩ , G8∞)	n/a
14	Van Munster 2016 [27]	Prospective cohort 144	(Pre)dialysis	Mean 65,2 (SD 12.0) Male 57%	VMS score ≥ 1 (0–4)	Frailty (FFI ∩ , ISAR-HP ∪ , GFI ~)	n/a
15	Van der Ven 2015 [21]	Retrospective cohort 5829	Hospital patients ≥ 65 years	*Not readmitted* Median 74 (69–79) Male 53.9% *Readmitted* Median 73 (68–79) Male 60.7%	Number of domains	Readmission	30 days
16	Van der Zanden 2021 [41]	Retrospective cohort 157	Gynecological surgical ≥ 70	Median 74 (IQR 71–79)	VMS score ≥ 1 (0–4)	Mortality Postoperative complications (Clavien–Dindo) Delirium Readmissions Change living situation	6 months 30 days
17	Warnier 2020 [23]	Retrospective cohort 2573	Community-dwelling hospital patients ≥ 70 years	Mean 78.8 (SD 6.3) Male 48.2%	VMS score ≥ 1 (0–4)	Mortality Readmission Length of stay Discharge destination Frailty (MFST-HP)**	30 day 120 day

*Emergency Department (ED)

†Percutaneous Coronary Intervention (PCI)

‡ST-segment elevation myocardial infarction (STEMI)

§Serious adverse events (SAEs)

||Short-VMS includes the following VMS questions: 1) memory problems (delirium), 2) history of acute confusional state or delirium (delirium), 3) unintentional weight loss (malnutrition), 4) help in ADL (physical impairment), 5) fall in last 6 months (Short-VMS)

□Clinical Frailty Scale (CFS)

∩ Fried Frailty Index (FFI)

∪ Identification Seniors At Risk Hospitalized Patients (ISAR-HP)

#Comprehensive Geriatric Assessment (CGA)

**Maastricht Frailty Screening Tool for Hospitalized patients (MFST-HP)

∞Geriatric 8 (G8)

^~^Groningen Frailty Indicator (GFI)

### Data extraction and analysis

Data were extracted by one researcher (FO) and were reviewed by a second researcher (MM). A standardized form was used to extract data from the included studies for assessment of study quality and evidence synthesis (Supplementary D). The following descriptive data were extracted: year of publication, language, country, study design, study setting, and study population. Participant demographics included number of participants, age, and sex. Regarding the VMS frailty instrument, we extracted information about how it was used as a screener, in particular the cut-off scores for identification of frailty. All outcomes and variables that were assessed in the studies for a relationship with VMS frailty instrument were recorded. If the VMS instrument was compared to other frailty measures of screening instruments, we reported and compared the measurement properties. We contacted the authors of studies who did not report data on agreement to supply additional information.

### Critical appraisal of individual sources of evidence

We performed a critical appraisal of the included studies. The QUIPS tool was used for observational (prognosis/screening) studies to assess the methodological quality (Supplementary E: Table E.1) [19]. This tool consists of six domains that should be critically appraised when evaluating validity and bias in studies of prognostic factors. Three reviewers independently performed the assessment, with disagreements resolved by discussion (FO, MM, and HM).

## Results

We identified 603 records through database searches, of which 188 in Pubmed, 391 in Embase, and 24 in Cinahl. We removed 169 duplicates (Fig. 1), and after title and abstract reading, 27 articles remained eligible. After full text selection, 17 of these 27 studies were included. [13, 14, 20–31] Articles were excluded because: 404 were not about the VMS instrument, 2 papers used VMS as an inclusion criterion, 2 papers reported on the implementation of VMS, 2 papers only studied the VMS delirium risk score, 1 study did not cover all four VMS domains, 1 paper only studied the individual domains and not an overall score, 3 were conference abstracts, 1 paper did not study VMS as frailty instrument or as predictor for adverse outcomes and 1 paper only reported crude incidence and no VMS overall score [10, 26, 32–38]. We checked the citations of included articles and found 53 records, of which no additional papers were eligible for inclusion either because they were already included papers or did not study the VMS.

The characteristics of included studies are shown in Table 1. All 17 studies were cohort studies, of which 7 were retrospective and 10 prospective. In total, 18,284 patients were included. The VMS frailty instrument was studied in a variety of hospital settings (i.e., different type of wards, or emergency department) and populations. The most frequent settings were hospitalized patients and the emergency department (*n* = 5; *n* = 5). A specific population studied more than in one study were pre-dialysis patients (*n* = 2) (Table 2).

**Table 2:**
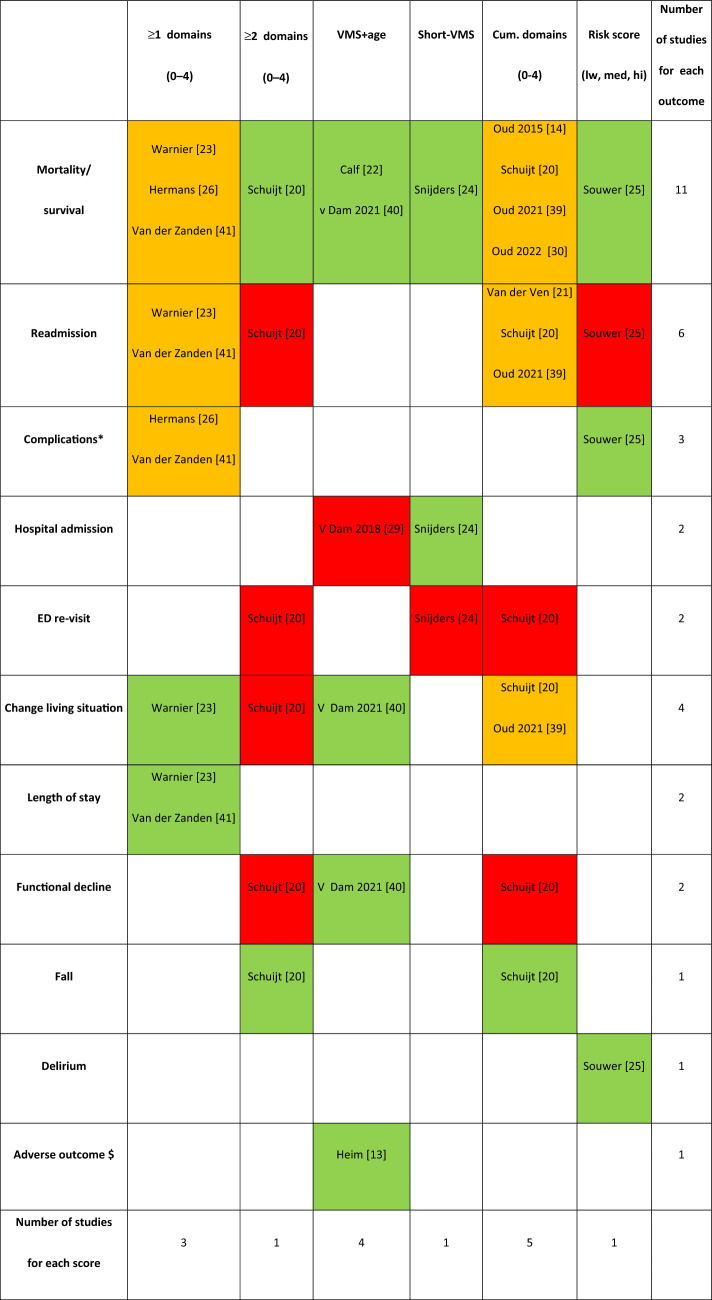
Outcomes used in studies and the number of studies significantly predictive and not predictive for the individual outcomes

Green: predictive; orange: conflicting results; red: not predictive

*Complications: vd Zanden et al.; postoperative complications (Clavien-Dindo), Hermans et al.; PCI-related SAEs (clinical death, target vessel failure, major bleeding, contrast induced kidney insufficiency and stroke) Souwer et al.; complication (surgical, non-surgical, cardiopulmonary, any)

$ Adverse outcome (functional decline, high-healthcare demand, death)

### Quality assessment and risk of bias

We used the QUIPS tool to assess the quality of included studies [19]. Twelve studies had a moderate risk of bias (Supplementary E, Table E.1), which may be explained by the fact that they were all cohort studies. We found five studies that had a high risk of bias, of which three retrospectively collected hospital data.

### Heterogeneity in the scoring of the Safety Management System (VMS) frailty instrument

Within the VMS frailty instrument, there is heterogeneity in the assessment of the domains malnutrition and physical impairment and in the overall VMS score.

#### Malnutrition: SNAQ or MUST

Within VMS, malnutrition can be scored with the Short Nutritional Assessment Questionnaire (SNAQ) or Malnutrition Universal Screening Tool (MUST) [15, 16]. Using SNAQ, a score of 2 indicates moderate malnutrition and a score of ≥ 3 severe malnutrition [15]. With the MUST, a score of 1 indicates moderate malnutrition and a score of ≥ 2 severe malnutrition [42]. Ten studies used the SNAQ score [13, 20, 26–30, 39, 43], three studies used the MUST score [21–23]. One multicentre study used both the MUST and SNAQ [25]. Snijders and colleagues only used one question of the SNAQ [24]. Two studies did not report if they used MUST or SNAQ [31, 40].

#### Physical impairment: eating or walking

The six-item Katz-ADL Index is used to assess functional status [17]. In the first version of the VMS, the question whether patients needed help with eating was mistakenly replaced with a question about help with walking. Later, this was corrected. However, two studies reported they used the question about walking, [14, 23] and eight studies reported they used the question about eating [13, 20, 25–28, 30, 39]. The other studies did not describe the individual Katz-ADL questions or used a shorter version of the Katz without this question [21, 24, 29, 31, 40].

#### Various VMS overall scores

In the included studies, seven different VMS scores were used (Table 1), namely:

1. The number of positive domains, and based on this number, a cut-off value was chosen (i.e., ≥ 1 VMS domains, ≥ 2 VMS domains). The most common cut-off scores used were one or more domains (*n* = 4) or two or more domains (*n* = 2) (Table 1) [13, 20, 23, 26–28].
2. VMS + age which classifies patients as frail if three or more domains were positive for patients aged 70–79, or one or more domains and aged 80 or over (*n* = 3, Table 1) [13, 22, 29, 40].
3. The “Short VMS” assesses all four domains, based on five questions of the VMS frailty instrument. A patient was classified as frail if they scored two out of five questions positive: history of delirium, memory problems, ADL dependency, weight loss, previous fall [24].
4. Six studies used cumulative scores without cut-off scores. One study composed a cumulative risk score of three categories: low risk (zero domains), medium risk (one or two domains), and high risk (three or four domains) for frailty [25]. Six studies added up the number of positive domains resulting in an ordinal score of 0–4 [14, 20, 21, 30, 31, 39] (Table 1).

To assess the value of the different VMS scores, we grouped the studies by the different scoring methods and reported the measurement properties if they were reported in the studies. Table 3 shows variation in sensitivity (i.e., true positive) for the different outcome measures. For example, the sensitivity for the outcome mortality is examined in three studies which varies between 57%–91% [22–24, 29]. Due to the small number of studies per VMS score and the heterogeneity in populations and outcomes, it was not possible to pool any of the data and perform meta-analysis.

**Table 3:** Comparison of measurement properties of different VMS scores

	Sens (%)	Spec (%)	PPV (%)	NPV (%)	AUC	Outcome
≥ 1 domains (0–4)						
Heim 2015 [13]	89	35	33	90	0.62	Adverse event
Van Munster 2016 [27]	77	67	57	83	0.76	Frailty (FFI)*
Warnier 2020 [23]	68	52	32	83	0.63	Discharge destination
	55;55	47;47	13;27	88;75	0.50;0.50	Readmission 30d; 120d
	91;84	49;50	8;15	99;67	0.76;0.71	Mortality 30d; 120d
≥ 2 domains (0–4)						
Heim 2015 [13]	67	66	42	84	0.66	Adverse event
Schuijt 2020 [20]			22 15	92 94		Fall Mortality
Van Loon 2017 [28]	90	38	78	60		Frailty (CGA†)
VMS + age (*Aged 70–80* ≥ *3; Aged* ≥ *80* ≥ *1)*						
Heim 2015 [13]	61	75	57	78	0.68	Adverse event
Calf 2020 [22]	57	71	41	82		Mortality
van Dam 2021 [40]	59 59 67	58 62 62			0.66 0.64 0.68	Mortality Functional decline Institutionalization
Short-VMS						
Snijders 2020 [24]	67	75	29	94	0.80	Mortality
VMS 0–4						
Schuijt 2020 [20]					0.67 0.49 0.56 0.58 0.52 0.65 0.49	Fall Functional decline Change living situation ED revisit Admission Mortality Composite outcome

*Fried Frailty Index (FFI)

†Comprehensive Geriatric Assessment (CGA)

### VMS instrument as frailty screener

#### Comparison to gold standard

Two studies, both performed in a population of end-stage renal disease patients, investigated the VMS frailty instrument and other frailty screening instruments compared with a gold standard for frailty [27, 28]. One study [27] used the number of impaired domains in the Comprehensive Geriatric Assessment (CGA) as the gold standard for diagnosing frailty [44]. The CGA is a multidisciplinary systematic method assessing a patient based on four domains: somatic, psychologic, physical function and, social domain. The other study [28] chose the Fried Frailty Index (FFI), assessing physical frailty, as frailty reference (Table 4) [45]. Compared to the FFI, the VMS with a score of ≥ 1 had a sensitivity of 77% and a VMS with score ≥ 2 had a sensitivity of 90% based on the CGA.

**Table 4:** Measurement properties of the VMS to assess frailty (based on CGA [28] or Fried Frailty Index (FFI) [27] compared to other frailty screening tools

	Sens (%)	Spec (%)	PPV (%)	NPV (%)	AUC
*Frailty compared with the golden standard Comprehensive Geriatric Assessment (CGA)* Van Loon 2017 [28]					
VMS* ≥ 2	90	38	78	60	
ISAR-HP†	72	79	91	48	
FFI‡	59	85	92	41	
G8§	92	26	79	53	
GFI||	74	52	82	40	
Frailty question	72	67	88	42	
*Frailty compared with the golden standard Fried Frailty Index (FFI)* Van Munster 2016 [27]					
VMS ≥ 1	77	67	57	83	0.76
GFI	89	57	54	90	0.83
ISAR-HP	83	77	67	89	0.89

*Safety Management System (VMS)

†Identification Seniors At Risk Hospitalized Patients (ISAR-HP),

‡Fried Frailty Index (FFI)

§ Geriatric 8 (G8)

||Groningen Frailty Indicator (GFI)

Table 4 shows the sensitivity of different frailty screening instruments with the FFI and the CGA as golden standard [27, 28]. Compared to other screening instruments that assess frailty, the screening instrument Geriatric 8 (G8) had the highest sensitivity (92%) based on the FFI, but had a high percentage of false positives. The Groningen Frailty Indicator (GFI) had the highest negative predictive value (NPV) (40%), meaning that 40% of the patients were misidentified as fit, when they could be frail.

#### Prevalence of frailty and agreement

Six papers reported the agreement between VMS and other screening instruments (i.e., VMS, Identification Seniors At Risk Hospitalized Patients (ISAR-HP), Geriatric 8 (G8), Groningen Frailty Index (GFI), Triage Risk Screening Tool (TRST); Maastricht Frailty Screening Tool-Hospitalized Patients (MFST-HP) and InterRAI) (Supplementary F, Table F.1) [13, 22, 23, 27–29]. The agreement between VMS and other frailty screening instruments ranged from 57 to 87%. One paper reported an adequate correlation between the CFS and VMS frailty screening (rSpearman = 0.55, 95% CI 0.40–0.62). [31] The percentage classified as frail by the VMS in the different studies varied from 34 to 88%.

### Adverse outcomes

In 14 papers, adverse health outcomes were studied as a derivate of frailty. Table 2 gives an overview of all assessed outcomes and shows that the VMS was predictive for most of the assessed outcomes, with mortality the most common outcome examined [20, 22–26, 30, 39–41, 43]. Conflicting results were found for the outcome hospital admission, readmission, complications, change in living situation, functional decline, and mortality. VMS was not predictive for a revisit to the emergency department. Furthermore, the way adverse outcomes assessed in the 14 studies were heterogeneous. First, the definitions of adverse outcomes, complications, and adverse events varied (Table 1).

And second, there was variation in methodology. Eight papers reported odds ratios (ORs), hazard ratios (HRs) or the relative risk (RR) of VMS scores for adverse health outcomes (Supplementary G, Table G.1). [13, 14, 23–26, 39, 41] [14, 24, 26, 38]

In Supplementary H (Table H.1), the values from the individual studies are reported. Five papers studied the measurement properties of the VMS for adverse outcomes [13, 20, 22–24]. Measurement properties of the frailty instruments varied greatly between the studies [13, 22, 23].

## Discussion

Our review aimed first to compare the VMS frailty instrument as a frailty screener in older patients in different hospital settings with existing frailty instruments and second to provide an overview of the available evidence to be able to make a recommendation for future use.

We revealed three main results. First, even though the VMS frailty instrument has not been developed as a frailty screener, its measurement properties are comparable with other existing frailty instruments, with the highest sensitivity and specificity of VMS for frailty of 90% and 67%, respectively. Second, all the various reported VMS scores, both continuous and cut-off scores, have similar measurement properties compared with other frailty instruments. Third, the VMS is associated with health outcomes, such as falls, delirium, longer length of hospital stay, and adverse events in older patients. The VMS might be associated with hospital admission, readmission, complications, functional decline, change in living situation, and mortality. The VMS is not associated with ED re-visits.

Two studies compared the VMS with gold standards (i.e., FFI and CGA) and assessed the sensitivity for frailty. The highest sensitivity was reported for a higher VMS cut-off score (two or more domains positive). This seems illogical (because with lower cut-off scores, more patients are expected to be classified as frail), but may be explained by the different gold standard tests used in these studies; the FFI focusses on physical frailty, whereas the CGA has a broader scope [44, 45].

Our review showed that the VMS frailty instrument might be used as a frailty screener in a variety of hospital settings and populations, as all various VMS scores have similar mediocre measurement properties (i.e., in terms of sensitivity, specificity, NPV, and PPV) compared to other frailty instruments. We believe that it is difficult to develop a frailty instrument with higher values for sensitivity without compromising too much on specificity. Frailty is a multifactorial, heterogeneous concept with many phenotypes which is difficult to capture in a quick and simple screening tool with good measurement properties [28]. The heterogeneity of the frailty concept is illustrated by the moderate agreement (63–75%) and large differences in percentage of patients screened as frail (16–88%) by the VMS and other frailty instruments shown in Supplementary F. The moderate agreement between various frailty screening tools is a well-known issue that is also found in previous papers [46]. Besides the heterogeneity of the frailty concept, there is another factor why in this review the VMS and other frailty instrument show moderate agreement and large differences in percentage of patients screened as frail. This is because of the wide range of settings and populations we included.

Most included studies did not examine the value of the VMS instrument as a screener for frailty, but the predictive value for adverse health outcomes as a derivative for frailty. The VMS frailty instrument was gradually implemented from 2009 in all Dutch hospitals to take preventive measures if necessary; this may have affected the results, as adverse outcomes may have been reduced. Examples of tailored preventive measures that the VMS frailty program recommends are physical therapy in case of high fall risk or consulting a dietician in case of undernutrition. However, this study showed that the VMS was predictive for delirium, falls, length of stay, and adverse outcomes; doubtful predictive for hospital admission, complications, readmission, change in living situation, functional decline, and mortality; and not predictive for ED re-visits. These conflicting results arise from the differences in the way the VMS frailty instrument was scored, outcomes were assessed and differences in populations. We were not able to determine which scoring method has the best predictive value due to the heterogeneity in the studies. Given the heterogeneity of frailty, it remains to be seen if a one size fits all cut-off is possible or even desirable.

A strength of our review is that this is the first review on the VMS frailty instrument for older patients. Also, in this study, we used broad inclusion criteria, allowing us to capture a wide range of information about the use of the VMS in research and clinical practice. Another strength is that we examined the quality of the evidence presented in each article which allows us to provide a high-level overview of the VMS frailty instrument and its usability.

However, there are also some limitations. Due to the heterogeneity of the available studies, it was impossible to compare studies comprehensively or perform meta-analysis. Also, most included studies examined adverse health outcomes as a derivate of frailty and preventive measures may have had an effect on these outcomes. However, performing controlled studies would not be ethical these days, as the VMS frailty instrument and its preventive measures are part of the current standard care in Dutch hospitals.

The overarching goal of screening for frailty is improving healthcare outcomes for frail older patients. This can be achieved by applying frailty screeners for different purposes, to prevent poor health outcomes by taking preventive measures, guide clinical decision-making, and advance care planning. These different purposes come with different requirements for screening instruments. Since this review showed that the VMS instrument performs as good (or bad) as other frailty instruments with various scores in a variety of populations and hospital settings, the VMS could potentially be the starting point of a ‘flexible frailty screening tool’. In light of this, we recommend to tailor frailty screening to the specific requirements of a setting and population due to the multifactorial nature of frailty and the heterogeneity of older hospital patients.

We will illustrate this with a few examples. The first example is risk stratification in treatment decisions. In shared decision-making, a cumulative frailty score gives the most information about the severity of frailty. Second, the setting in which patients are screened calls for tailor-made adjustments. For example, in the Emergency Department, a quick and simple tool, like a short version of the VMS frailty instrument (i.e., Short-VMS), is preferred. Third, different populations may ask for specific requirements. For example, in prehabilitation before a planned surgery, a screener that can identify specific risks in specific domains so that appropriate interventions can be deployed might be more helpful.

Another example of population-specific requirements is assessment of undernutrition in heart failure patients. In this population, it might not be reliable to assess nutritional status based on weight due to the variability in weight because of edema. Thus, in our opinion, there is no ‘one size fits all’ solution in screening for frailty and the way forward is adaptable frailty screening tools tailored to specific situations.

The VMS frailty instrument studied several specific sub-populations of older patients; the emergency department, colon surgery, gynecology, (pre)dialysis, burn injury patients, oncology and cardiothoracic surgery. [10] There are, however, other potentially frail populations in clinical practice that might benefit from implementing a tailored frailty instrument like the VMS to improve health care outcomes, for example patients with heart failure or COPD and in orthogeriatric patients.

## Conclusion

We found that the VMS frailty instrument is studied as a frailty screener in various populations and settings. The value of the VMS instrument as a frailty screener looks promising, the instrument has similar measurement properties compared to other frailty instruments and is a good predictor for adverse events. Our results also suggest that the scoring method of the VMS could be adapted to specific requirements of hospital settings, characteristics of populations, and aim of the screening.

### Supplementary Information

Below is the link to the electronic supplementary material.

Supplementary file1 (DOCX 138 kb)
