# Supplementary material for: Value of the Safety Management System (VMS) frailty instrument as a frailty screener in care for older hospital patients: a systematic review
Source: Eur Geriatr Med. 2024 Apr 26;15(3):609–20. doi: 10.1007/s41999-024-00957-4 (PMC11329526; doi:10.1007/s41999-024-00957-4)
Supplement: Supplementary file 1 — Supplementary file1 (DOCX 138 kb) [file 41999_2024_957_MOESM1_ESM.docx]

**Supplement A: VMS frailty instrument**

*Figure A.1: shows the VMS frailty instrument questions.*

*KATZ-ADL = Katz index of independence in activities of daily living; SNAQ =short nutritional assessment questionnaire; MUST= Malnutrition Universal Screening Tool; VMS = Dutch Safety Management System.*

*The VMS instrument as whole is not available nor validated in English. However three of the four domains are validated instruments that already exist; the fall risk question, the MUST or SNAQ and the Katz-ADL. We have translated the delirium risk question from Dutch to English.*


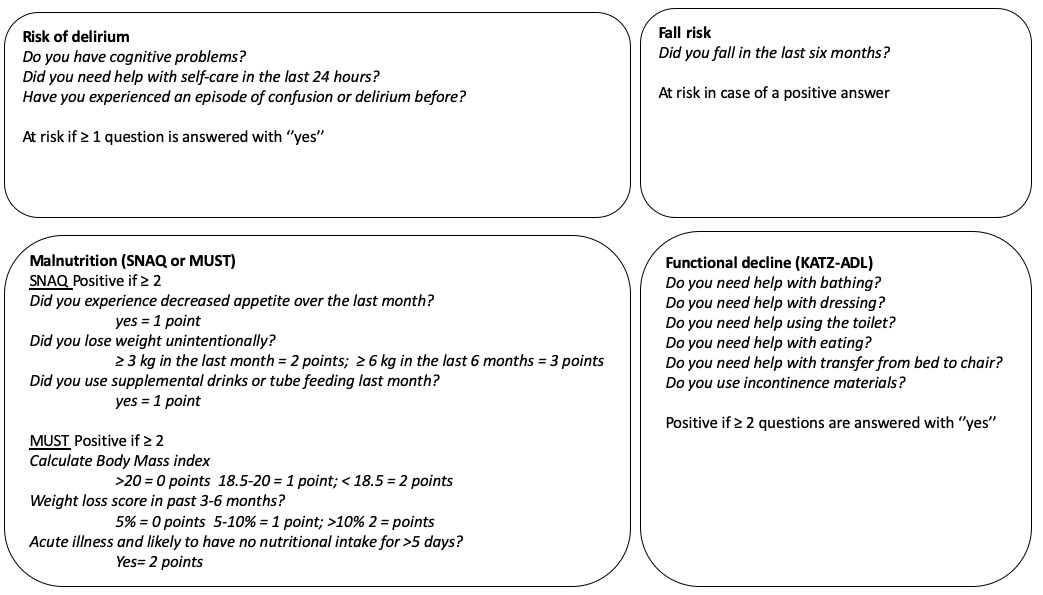


**Supplement B: Preferred Reporting Items for systematic reviews and Meta-Analyses (PRISMA) checklist**

Table B.1: shows a detailed description of the search strategy.

| **Section and Topic** | **Item #** | **Checklist item** | **Location where item is reported** |
| --- | --- | --- | --- |
| **TITLE** | | |  |
| Title | 1 | Identify the report as a systematic review. | Title and method |
| **ABSTRACT** | | |  |
| Abstract | 2 | See the PRISMA 2020 for Abstracts checklist. |  |
| **INTRODUCTION** | | |  |
| Rationale | 3 | Describe the rationale for the review in the context of existing knowledge. | Introduction |
| Objectives | 4 | Provide an explicit statement of the objective(s) or question(s) the review addresses. | Aim (introduction) |
| **METHODS** | | |  |
| Eligibility criteria | 5 | Specify the inclusion and exclusion criteria for the review and how studies were grouped for the syntheses. | Method: ‘’ selection criteria ‘ data extraction and analysis’ |
| Information sources | 6 | Specify all databases, registers, websites, organisations, reference lists and other sources searched or consulted to identify studies. Specify the date when each source was last searched or consulted. | Method: ‘Search strategy and selection criteria’ |
| Search strategy | 7 | Present the full search strategies for all databases, registers and websites, including any filters and limits used. | Supplement 2 |
| Selection process | 8 | Specify the methods used to decide whether a study met the inclusion criteria of the review, including how many reviewers screened each record and each report retrieved, whether they worked independently, and if applicable, details of automation tools used in the process. | Method: ‘Search strategy and selection criteria’ |
| Data collection process | 9 | Specify the methods used to collect data from reports, including how many reviewers collected data from each report, whether they worked independently, any processes for obtaining or confirming data from study investigators, and if applicable, details of automation tools used in the process. | Method: ‘ data extraction and analysis |
| Data items | 10a | List and define all outcomes for which data were sought. Specify whether all results that were compatible with each outcome domain in each study were sought (e.g. for all measures, time points, analyses), and if not, the methods used to decide which results to collect. | Method: ‘ data extraction and analysis |
|  | 10b | List and define all other variables for which data were sought (e.g. participant and intervention characteristics, funding sources). Describe any assumptions made about any missing or unclear information. | Method: ‘ data extraction and analysis |
| Study risk of bias assessment | 11 | Specify the methods used to assess risk of bias in the included studies, including details of the tool(s) used, how many reviewers assessed each study and whether they worked independently, and if applicable, details of automation tools used in the process. | Method: critical appraisal of individual sources of evidence |
| Effect measures | 12 | Specify for each outcome the effect measure(s) (e.g. risk ratio, mean difference) used in the synthesis or presentation of results. | Method: ‘ data extraction and analysis |
| Synthesis methods | 13a | Describe the processes used to decide which studies were eligible for each synthesis (e.g. tabulating the study intervention characteristics and comparing against the planned groups for each synthesis (item #5)). | Method: ‘ data extraction and analysis |
|  | 13b | Describe any methods required to prepare the data for presentation or synthesis, such as handling of missing summary statistics, or data conversions. | Na |
|  | 13c | Describe any methods used to tabulate or visually display results of individual studies and syntheses. |  |
|  | 13d | Describe any methods used to synthesize results and provide a rationale for the choice(s). If meta-analysis was performed, describe the model(s), method(s) to identify the presence and extent of statistical heterogeneity, and software package(s) used. | Na |
|  | 13e | Describe any methods used to explore possible causes of heterogeneity among study results (e.g. subgroup analysis, meta-regression). | Na |
|  | 13f | Describe any sensitivity analyses conducted to assess robustness of the synthesized results. | Na |
| Reporting bias assessment | 14 | Describe any methods used to assess risk of bias due to missing results in a synthesis (arising from reporting biases). | Method: critical appraisal of individual sources of evidence |
| Certainty assessment | 15 | Describe any methods used to assess certainty (or confidence) in the body of evidence for an outcome. | Method: ‘ data extraction and analysis |
| **RESULTS** | | |  |
| Study selection | 16a | Describe the results of the search and selection process, from the number of records identified in the search to the number of studies included in the review, ideally using a flow diagram. | Flow diagram |
|  | 16b | Cite studies that might appear to meet the inclusion criteria, but which were excluded, and explain why they were excluded. | Results |
| Study characteristics | 17 | Cite each included study and present its characteristics. | Table 1 |
| Risk of bias in studies | 18 | Present assessments of risk of bias for each included study. | Results: risk of bias, Supplement 4, Table S4 |
| Results of individual studies | 19 | For all outcomes, present, for each study: (a) summary statistics for each group (where appropriate) and (b) an effect estimate and its precision (e.g. confidence/credible interval), ideally using structured tables or plots. | Na |
| Results of syntheses | 20a | For each synthesis, briefly summarise the characteristics and risk of bias among contributing studies. | Na |
|  | 20b | Present results of all statistical syntheses conducted. If meta-analysis was done, present for each the summary estimate and its precision (e.g. confidence/credible interval) and measures of statistical heterogeneity. If comparing groups, describe the direction of the effect. | Na |
|  | 20c | Present results of all investigations of possible causes of heterogeneity among study results. | Na |
|  | 20d | Present results of all sensitivity analyses conducted to assess the robustness of the synthesized results. | Na |
| Reporting biases | 21 | Present assessments of risk of bias due to missing results (arising from reporting biases) for each synthesis assessed. | Na |
| Certainty of evidence | 22 | Present assessments of certainty (or confidence) in the body of evidence for each outcome assessed. | Na |
| **DISCUSSION** | | |  |
| Discussion | 23a | Provide a general interpretation of the results in the context of other evidence. | Discussion: comparison to literature |
|  | 23b | Discuss any limitations of the evidence included in the review. | Discussiion: strengths and limitations |
|  | 23c | Discuss any limitations of the review processes used. | Discussiion: strengths and limitations |
|  | 23d | Discuss implications of the results for practice, policy, and future research. | Discussion: recommendations |
| **OTHER INFORMATION** | | |  |
| Registration and protocol | 24a | Provide registration information for the review, including register name and registration number, or state that the review was not registered. | Review was not registered |
|  | 24b | Indicate where the review protocol can be accessed, or state that a protocol was not prepared. | No protocol was prepared |
|  | 24c | Describe and explain any amendments to information provided at registration or in the protocol. | Na |
| Support | 25 | Describe sources of financial or non-financial support for the review, and the role of the funders or sponsors in the review. | No funding |
| Competing interests | 26 | Declare any competing interests of review authors. | No competing interest |
| Availability of data, code and other materials | 27 | Report which of the following are publicly available and where they can be found: template data collection forms; data extracted from included studies; data used for all analyses; analytic code; any other materials used in the review. | Na |

**Supplement C: search strategy**

Medline (Pubmed)

("Aged"[Mesh] OR elder*[tiab] OR "older adult*"[tiab] OR "older patient*"[tiab] OR "older people"[tiab] OR "old people"[tiab] OR "old person*"[tiab] OR "older person*"[tiab] OR "old adult*"[tiab] OR "older adult*"[tiab] OR geriatric*[tiab] OR "old age"[tiab] OR "over 65"[tiab] OR frail[tiab] OR frailty[tiab]) 
AND 
(vms[tiab] OR veiligheidsmanagementsysteem[tiab] OR "safety management system*"[tiab] OR "safety management program*"[tiab] OR "hospital safety program*"[tiab])
AND 
("2008/01/01"[pdat] : "3000"[pdat])

Embase

('aged'/exp OR (elder* OR "older adult*" OR "older patient*" OR "older people" OR "old people" OR "old person*" OR "older person*" OR "old adult*" OR "older adult*" OR geriatric* OR "old age" OR "over 65" OR frail OR frailty):ab,ti) 
AND 
(vms OR veiligheidsmanagementsysteem OR "safety management system*" OR "safety management program*" OR "hospital safety program*"):ab,ti
AND 
[2008-2023]/py

CINAHL EBSCO

TX (elder* OR "older adult*" OR "older patient*" OR "older people" OR "old people" OR "old person*" OR "older person*" OR "old adult*" OR "older adult*" OR geriatric* OR "old age" OR "over 65" OR frail OR frailty)

AND

TX (vms OR veiligheidsmanagementsysteem OR "safety management system*" OR "safety management program*" OR "hospital safety program*")

**Supplement D: data extraction items**

| **General information** |
| --- |
| Author, year |
| Country |
| Language |
| **Study characteristics** |
| design |
| setting |
| **Participant characteristics** |
| Number of patients |
| Mean/median age |
| Gender |
| **VMS** |
| how VMS was scored |
| Comparison to other frailty measure |
| **methodology** |
| Analysis methods |
| **Outcomes** |
| Any reported outcome |
| **Other:** key conclusion of the authors of the included study, conclusions / comments |

**Supplement E: Quality assessment**

*Table E.1: Risk of bias in included studies*

|  | **Reference** | **Study participation** | **Study attrition** | **Prognostic factor measurement** | **Outcome measurement** | **Study confounding** | **Statistical analysis and reporting** |
| --- | --- | --- | --- | --- | --- | --- | --- |
| 1. | Calf 2020 | *Moderate* | *Low* | *Low* | *Low* | *Low* | *Low* |
| 2. | Cords 2023 | *Moderate* | *Low* | *Moderate* | *Low* | *Moderate* | *Low* |
| 3. | Heim 2015 | *Moderate* | *Moderate* | *Moderate* | *Moderate* | *Low* | *Moderate* |
| 4. | Hermans 2019 | *Moderate* | *Moderate* | *Low* | *Low* | *Low* | *Low* |
| 5. | Oud 2015 | *Moderate* | *Low* | *Low* | *Low* | *Moderate* | *Low* |
| 6. | Oud 2021 | *Moderate* | *Low* | *Moderate* | *Low* | *Moderate* | *Low* |
| 7. | Oud 2022 | *Low* | *Low* | *Low* | *Low* | *High* | *Low* |
| 8. | Schuijt 2020 | *Moderate* | *Moderate* | *Moderate* | *Low* | *Moderate* | *Low* |
| 9. | Snijders 2020 | *High* | *Moderate* | *Moderate* | *Low* | *Moderate* | *Low* |
| 10. | Souwer 2019 | *Moderate* | *Low* | *Low* | *Low* | *Low* | *Low* |
| 11. | Van Dam 2018 | *High* | *Low* | *Low* | *Low* | *Low* | *Moderate* |
| 12. | Van Dam 2021 | *Moderate* | *High* | *Moderate* | *Moderate* | *Moderate* | *Low* |
| 13. | Van Loon 2017 | *Moderate* | *Moderate* | *Low* | *Low* | *Low* | *Low* |
| 14. | Van Munster 2016 | *Moderate* | *Moderate* | *Low* | *Low* | *Low* | *Low* |
| 15. | Van der Ven 2015 | *High* | *High* | *High* | *Moderate* | *Moderate* | *Low* |
| 16. | Van der Zanden 2021 | *High* | *Moderate* | *Moderate* | *Low* | *Low* | *Low* |
| 17. | Warnier 2020 | *Moderate* | *Moderate* | *Moderate* | *Moderate* | *Low* | *Low* |

**Supplement F: Agreement of VMS with other frailty instruments**

*Table F.1: Agreement of VMS with other frailty instrument and percentage patients screened as frail. Agreement is expressed in percentages and Cohens kappa coefficient (k). Correlation is expressed in* rSpearman

| **Reference** | **Frail (%)** | **Agreement** | **Correlation** |
| --- | --- | --- | --- |
| **Calf 2020** | | |  |
| VMS*+age | 53 |  |  |
| Frailty question nurse | 62 | 63%;K= 0.31 |  |
| Frailty question physician | 65 | 63%;K= 0.32 |  |
| **Cords 2023** | | |  |
| CFS | 37 |  | 0.55, 95% CI 0.40–0.62 |
| **Heim 2015** | | |  |
| VMS ≥1 | 72 | 64%;K=0.26 |  |
| VMS­ ≥2 | 43 | 66%;K=0.33 |  |
| VMS+age | 34 | 66%;K=0.32 |  |
| ISAR-HP† | 57 |  |  |
| **Van Dam 2018** | | |  |
| VMS +age | 38 |  |  |
| ISAR-HP | 45 | 72%,K= 0.43 |  |
| InterRAI | 22 | 75%,K= 0.41 |  |
| APOP | 19 | 77%,K=0.44 |  |
| **Van Loon 2017** | | |  |
| VMS≥2 | 82 |  |  |
| ISAR-HP | 60 | 66%;K=0.24 |  |
| FFI‡ | 48 | 61%;K=0.27 |  |
| G8§ | 88 | 87%; K=0.51 |  |
| GFI\|\| | 67 | 77%;K=0.40 |  |
| Frailty question | 63 | 57%;K=-0.02 |  |
| CGA** ≥2 | 75 | 75%;K=0.31 |  |
| **Van Munster 2016** | | |  |
| VMS≥1 | 49 |  |  |
| GFI | 60 | 73%; K=45 |  |
| ISAR-HP | 45 | 73%; K=45 |  |
| FFI | 35 | 71%; K=41 |  |
| **Warnier 2020** | | |  |
| VMS≥1 | 53 |  |  |
| MFST-HP# | 16 | 63%;K=0.29 |  |

*^*^ Safety management system (VMS)*

† *Identification Seniors At Risk Hospitalized Patients (ISAR-HP)*

‡ *Fried Frailty Index (FFI)*

§ *Geriatric 8 (G8)*

|| *Groningen Frailty Indicator (GFI)*

** *Comprehensive Geriatric Assessment (CGA)*

*# Maastricht Frailty Screening Tool for Hospitalized patients (MFST-HP)*

*Clinical Frailty Scale (CFS)*

**Supplement G: Measurement properties of VMS scores compared with other frailty instruments**

*Table G.1: Measurement properties of VMS scores compared with other frailty instruments to predict adverse health outcomes. Bold type indicates highest value for specific outcome*

|  | | **Sens (%)** | **Spec (%)** | **PPV (%)** | **NPV (%)** | **AUC** | **Outcome** |
| --- | --- | --- | --- | --- | --- | --- | --- |
| **Hospital patients** | | | | | | | |
| **Heim 2015** | | | | | | | |
|  | |  |  |  |  |  | Adverse event |
| VMS ≥1 | | **89** | 35 | 33 | **90** | 0.62 |  |
| VMS­ ≥2 | | 67 | 66 | 42 | 84 | 0.66 |  |
| VMS+age | | 61 | **75** | **57** | 78 | **0.68** |  |
| ISAR-HP^2^ | | 73 | 49 | 34 | 83 | 0.61 |  |
| **ED patients** | | | | | | | |
| **Calf 2020** | | | | | | | |
|  | |  |  |  |  |  | Mortality |
| VMS^1^ +age | | 57 | **71** | **41** | 82 | - |  |
| Frailty question nurse | | 86 | 46 | 37 | 90 | - |  |
| Frailty question physician | | **94** | 46 | 39 | **96** | - |  |
| **Schuijt 2020** | | | | | | | |
|  |  |  |  |  |  |  | Fall |
| VMS-0-4 |  | - | - | - | - | 0.67 |  |
|  |  |  |  |  |  |  | Functional decline |
| VMS-0-4 |  | - | - |  | - | 0.49 |  |
|  |  |  |  |  |  |  | Change living situation |
| VMS-0-4 |  | - | - | - | - | 0.56 |  |
|  |  |  |  |  |  |  | ED revisit |
| VMS-0-4 |  | - | - | - | - | 0.58 |  |
|  |  |  |  |  |  |  | Admission |
| VMS-0-4 |  | - | - | - | - | 0.52 |  |
|  |  |  |  |  |  |  | Mortality |
| VMS-0-4 |  | - | - | - | - | 0.65 |  |
|  |  |  |  |  |  |  | Composite outcome |
| VMS-0-4 |  | - | - | - | - | 0.49 |  |
| VMS ≥2 |  |  |  |  |  |  | Fall |
|  |  | - | - | 0.22 | 0.92 | - |  |
| VMS ≥2 |  |  |  |  |  |  | Mortality |
|  |  | - | - | 0.15 | 0.94 | - |  |
| **Snijders** **2020** | | | | | | | |
|  | |  |  |  |  |  | Mortality |
| Short-VMS | | 67 | 75 | 29 | 94 | 80 |  |
| **Community dwelling hospital patients** | | | | | | | |
| **Warnier 2020** | | | | | | | |
|  | |  |  |  |  |  | Discharge destination |
| VMS≥1 | | **68** | 52 | 32 | **83** | 0.63 |  |
| MFST-HP | | 25 | **87** | 39 | 77 | 0.63 |  |
|  | |  |  |  |  |  | Readmission30d;120d |
| VMS≥1 | | **55;55** | 47;47 | **13;27** | **88;75** | **0.50**;0.50 |  |
| MFST-HP | | 14;14 | **84;83** | 11;23 | 87;74 | 0.49;0.50 |  |
|  | |  |  |  |  |  | Mortality 30d;120d |
| VMS≥1 | | **91;84** | 49;50 | 8;15 | **99**;67 | **0.76;0.71** |  |
| MFST-HP | | 37;33 | **85;86** | **10;20** | 97;**92** | 0.69;68 |  |

*^1^ Safety management system (VMS)*

^2^ *Maastricht Frailty Screening Tool for Hospitalized Patients (MFST-HP)*

**Supplement H: Predictive value**

*Table H.1: Odds ratio (OR), Hazard Ratio (HR) and Relative Risk (RR) of Safety Management System (VMS) scores for adverse/negative health outcomes*

| **Paper & VMS-score** | **OR/HR/RR (95% CI)** | **Outcome** |
| --- | --- | --- |
| **Hospital patients** | | |
| **Heim 2015** | | |
| VMS ≥1 | RR 3.36 (2.28-4.95) | Adverse event |
| VMS ≥2 | RR 2.77 (2.18-3.52) | Adverse event |
| VMS+age | RR 2.60 (2.15-3.15) | Adverse event |
| **Oud 2015** | | |
| VMS 0 | Ref. | Mortality |
| 1 | OR 1.6 (0.8-3.2) |  |
| 2 | OR 2.3 (1.1-4.6) |  |
| 3 | OR 3.4 (1.6-7.1) |  |
| 4 | OR 8.6 (3.5-21.2) |  |
| **Oud 2022** | | |
| VMS 0 | Ref. | Mortality |
| 1 | OR 2.6 (1.8-3.7) |  |
| 2 | OR 5.4 (3.9-7.6) |  |
| 3 | OR 6.7 (4.7-9.5) |  |
| 4 | OR 9.8 (6.2-15.3) |  |
| **Patients who underwent PCI for STEMI** | | |
| **Hermans** 2019 | | |
| VMS ≥1(0-4) | OR 2.9 (1.1–7.9)  OR 9.6 (1.6–56.9) | PCI adverse event  Mortality |
| **Geriatric inpatients** | | |
| **Oud 2021** | | |
| VMS 0 | Ref. | Readmission |
| 1 | OR 3.2 (0.4-28.5) |  |
| 2 | OR 1.2 (0.1-10.3) |  |
| 3 | OR 1.5 (0.2-13.3) |  |
| 4 | OR 1.5 (0.2-13.8) |  |
| VMS 0 | Ref | Mortality 3 months; 12 months |
| 1 | OR 0.4 (0.1-1.7); OR 0.7 (0.2-2.8) |  |
| 2 | OR 0.5 (0.1-1.7); OR 1.0 (0.3-3.5) |  |
| 3 | OR 0.7 (0.2-2.4); OR 1.2 (0.4-4.8) |  |
| 4 | 0.8 (0.2-2.9); OR 1.1 (0.3-3.7) |  |
| VMS 0 | Ref | Institutionalization |
| 1 | OR 4.3 (0.5-36.3) |  |
| 2 | OR 5.4 (0.7-45.5) |  |
| 3 | OR 6.1 (0.7-50.0) |  |
| 4 | OR 12.1 (1.4-101.7) |  |
| VMS ≥1 | OR 1.6 (0.2-13.7) | Readmission |
|  | OR 0.6 (0.2-2.0) | Mortality 3 months |
|  | OR 1.1 (0.3-3.7) | Mortality 12 months |
|  | OR 6.4 (0.8-51.3) | Institutionalization |
| **ED patients** | | |
| **Snijders** **2020** | | |
| Short-VMS | OR 2.26 (1.32-3.86)  HR -0.80 (0.43-1.51)  HR 2.48 (1.31-4.71) | Admission  ED-revisit  Mortality |
| **Colorectal cancer surgery** | | |
| **Souwer 2019** | | |
| VMS Low  Medium  High | Ref  OR 1.8 (1.2–2.7)  OR 2.4 (1.0–5.5) | Complications |
| VMS Low  Medium  High | ref  OR 2.5 (0.9-10.4)  OR 2.9 (1.3–6.4) | Delirium |
| VMS Low  Medium  High | ref  OR 2.7 (1.6–4.5)  OR 2.5 (0.9-6.9) | Discharge not to home |
| VMS Low  Medium  High | ref  OR 1.7 (0.9–3.0)  OR 2.1 (0.6–7.0) | Readmission |
| VMS Low  Medium  High | ref  HR 1.9 (1.1-3.5)  HR 8.7 (4.0-19.2) | Survival |
| **Gynecological surgical** | | |
| **Van der Zanden 2021** | | |
| VMS ≥1 | OR 1.18 (﻿0.49–2.82) | Postoperative complications |
| **Community dwelling hospital patients** | | |
| **Warnier 2020** | | |
| VMS ≥1 | RR 1.85 (1.60-2.14)  RR 1.09 (0.89-1.34); 1.06 (0.93-1.21)  RR 8.97 (4.71-17.10); 4.53 (3.26-6.30) | Discharge destination  Readmission 30d;120d  Mortality 30d;120d |
